# Dilemma of Reporting Incidental Findings in Newborn Screening Programs for SCID: Parents’ Perspective on Ataxia Telangiectasia

**DOI:** 10.3389/fimmu.2019.02438

**Published:** 2019-11-06

**Authors:** Maartje Blom, Michiel H. D. Schoenaker, Myrthe Hulst, Martine C. de Vries, Corry M. R. Weemaes, Michèl A. A. P. Willemsen, Lidewij Henneman, Mirjam van der Burg

**Affiliations:** ^1^Department of Pediatrics, Leiden University Medical Centre, Leiden, Netherlands; ^2^Department of Pediatric Neurology, Radboudumc Amalia Children's Hospital and Donders Institute for Brain, Cognition and Behavior, Radboud University Medical Center, Nijmegen, Netherlands; ^3^Department of Biologicals, Innovation and Screening, National Institute for Public Health and the Environment, Bilthoven, Netherlands; ^4^Department of Medical Ethics and Health Law, Leiden University Medical Center, Leiden, Netherlands; ^5^Department of Pediatrics, Radboudumc Amalia Children's Hospital, Radboud University Medical Center, Nijmegen, Netherlands; ^6^Department of Clinical Genetics, Amsterdam Reproduction & Development Research Institute, Vrije Universiteit Amsterdam, Amsterdam UMC, Amsterdam, Netherlands

**Keywords:** ataxia telangiectasia, A-T, newborn screening, severe combined immunodeficiency, SCID, incidental finding, parents' perspective, questionnaire

## Abstract

**Background:** Ataxia Telangiectasia (A-T) is a severe DNA repair disorder that leads to a broad range of symptoms including neurodegeneration and a variable immunodeficiency. A-T is one of the incidental findings that accompanies newborn screening (NBS) for severe combined immunodeficiency (SCID), leading to an early diagnosis of A-T at birth in a pre-symptomatic stage. While some countries embrace all incidental findings, the current policy in the Netherlands on reporting untreatable incidental findings is more conservative. We present parents' perspectives and considerations on the various advantages vs. disadvantages of early and late diagnosis of A-T.

**Methods:** A questionnaire was developed and sent to 4,000 parents of healthy newborns who participated in the Dutch SONNET-study (implementation pilot for newborn screening for SCID). The questionnaire consisted of open-ended and scale questions on advantages and disadvantages of early and late diagnosis of A-T. To address potential bias, demographic characteristics of the study sample were compared to a reference population.

**Results:** A total of 664 of 4,000 parents sent back the questionnaire (response rate 16.6%). The vast majority of parents (81.9%) favored early diagnosis of A-T over late diagnosis. Main arguments were to avoid a long period of uncertainty prior to diagnosis and to ensure the most optimal clinical care and guidance from the onset of symptoms. Parents who favored late diagnosis of A-T stated that early diagnosis would not lead to improved quality of life and preferred to enjoy the asymptomatic “golden years” with their child. The majority of parents (81.1%) stated that they would participate in newborn screening for A-T if a test was available.

**Conclusions:** Reporting untreatable incidental findings remains a disputed topic worldwide. Although the current policy in the Netherlands is not to report untreatable incidental findings, unless the health advantage is clear, the majority of parents of healthy newborns are in favor of an early A-T diagnosis in the pre-symptomatic phase of the disorder. Our results as well as other studies that showed support for the screening of untreatable disorders may serve as valuable tools to inform policymakers in their considerations about NBS for untreatable disorders.

## Introduction

In the last years, newborn bloodspot screening (NBS) for severe combined immunodeficiency (SCID) has been introduced in several screening programs worldwide (1–3). NBS for SCID is based on the detection of T-cell receptor excision circles (TRECs) in dried blood spots. TRECs are formed during the T-cell receptor rearrangement, therefore serving as a biomarker for newly formed T-lymphocytes. SCID patients do not have (functional) T-cells and therefore lack TRECs (4). Several studies have shown that NBS for SCID is accompanied by a high number of incidental findings. Low/absent TRECs can also be identified in neonates with T-cell impairment syndromes (such as DiGeorge Syndrome, Down Syndrome or Ataxia Telangiectasia), newborns with T-cell impairment secondary to other neonatal conditions or patients with idiopathic lymphocytopenia (1, 5, 6). The relatively high number of incidental findings is met with hesitations by policy makers responsible for making decisions with regard to implementation of SCID in NBS programs. However, these infants with non-SCID lymphopenia disorders do seem to benefit from early detection and treatment, for example by the prevention and reduction of infections by antibiotic prophylaxes and protective measures (7). In addition, possible harm by receiving life attenuated rotavirus or BCG vaccines can be avoided (8). There are however, untreatable conditions with low TRECs that present asymptomatic at birth and for which health benefits by early detection remain disputable. A key example of these untreatable conditions is Ataxia Telangiectasia (A-T).

A-T is a rare, autosomal recessively inherited disorder caused by mutations in the *Ataxia Telangiectasia Mutated* (*ATM*) gene. This DNA repair disorder leads to a combination of systemic and neurological symptoms, including progressive ataxia, ocular telangiectasias, predisposition to malignancies and a variable immunodeficiency (9). Patients with classic A-T are asymptomatic in the first year of life, but progressive symptoms will develop shortly after. The prevalence of A-T is estimated to be between 1 in 40,000 and 1 in 100,000 live births (9). A-T is a complex disease to diagnose as clinical presentation and/or laboratory findings vary between patients. A curative treatment for A-T is not yet available, and most patients with the classic form of the disease die before the age of 30 years (9). Optimal symptomatic treatment in the setting of a dedicated and experienced multidisciplinary team of health care professionals is of great importance (10). Of note, heterozygous carriers of a pathogenic *ATM* mutation, i.e., the parents of the newborn that underwent NBS, have a slightly decreased life expectancy and increased risk of developing cancer, especially breast cancer (11, 12). This implies that NBS for SCID might reveal a health risk for family members of the screened newborn in addition to risks for the newborn itself.

A-T was first described as an incidental finding to NBS for SCID in 2013 in California (13). Retrospective analysis of NBS cards of A-T patients showed that not all A-T patients present with low TRECs at birth (13, 14). However, no significant associations could be identified between the newborn TREC numbers and phenotypic clinical and laboratory features of A-T (such as age at presentation with neurological symptoms, total CD3+ T-cell counts or time between symptom-onset and diagnosis). Since then, multiple NBS programs with different assays and cut-off values have identified A-T patients based on low TRECs over the last few years [California *n* = 5 (3), France *n* = 1 (6), Sweden *n* = 1 (5)].

NBS for SCID based on TREC-quantification is intended to identify SCID patients at birth in order to enable early diagnosis and treatment of an otherwise fatal disorder. Conventional follow-up diagnostics after abnormal TREC results consist of flow cytometry and genetic confirmation of the underlying mutation. By adding the *ATM* gene in follow-up gene panels, NBS programs engage in an active search for A-T patients with the additional chance of identifying carriers of *ATM* mutations. While the reporting of clinically relevant and treatable (incidental) disorders is undisputed in the field of (neonatal) screening, the current policy on reporting untreatable (incidental) disorders remains controversial. The Wilson and Jungner screening criteria (1968) guide toward screening for treatable disorders. In addition, the Health Council of the Netherlands states that NBS for untreatable disorders and reporting of untreatable incidental findings would not be in the immediate health interest of the child (15). With these considerations in mind and based on expert opinions that question the added value of early diagnosis in A-T patients, Dutch experts decided to exclude the *ATM* gene from the NBS follow-up gene panel. There were, however, two major conditions in this follow-up produce in the interest of potential A-T patients. First, in the case of low TRECs/T-cells without a confirmed underlying genetic defect but with an indication for hematopoietic stem cell transplantation (HSCT), *ATM* mutations have to be ruled out before starting with conditioning regimes. Second, in the case of idiopathic T-cell lymphocytopenia (without genetic diagnosis) and no indication for HSCT, the newborn will be enrolled in out-patient clinical follow-up visits. If any clinical symptoms matching A-T start to occur, additional diagnostics (*ATM* gene analysis) will be initiated immediately. This follow-up protocol ensures that during the Dutch implementation pilot for NBS for SCID (SONNET-study, www.sonnetstudie.nl) untreatable incidental findings will not be reported and A-T will not be an incidental finding to NBS for SCID in the Netherlands.

The perspective of parents as key stakeholders in NBS is of great value in policymaking. The aim of this study is therefore to gain insight into parents' perspectives about the early detection of A-T. Empirical data on the views of parents on early detection of A-T will provide insight into the public acceptance of untreatable incidental findings to NBS.

## Methods

### Study Population and Procedure

The study encompasses a cross-sectional survey study amongst parents of healthy newborns. A questionnaire was sent to 4000 Dutch parents of healthy newborns. Only parents from the pilot-provinces Utrecht, Gelderland and Zuid-Holland who participated in the SONNET-study were invited to participate (www.sonnetstudie.nl). In order to participate in the SONNET-study, parents have to express verbal consent when the heel prick is performed. If parents object to the SONNET-study and with that NBS for SCID, this was noted on the blood spot card and registered in the screening laboratories. Parents who objected to participation in the SONNET-study or the entire NBS program were not invited for this survey study. The questionnaire focused on a potential incidental finding of NBS for SCID, therefore parents were approached 8–10 weeks after their child received the heel prick in the hope information about NBS could still be recalled. Questionnaires could not be sent out earlier as parents with abnormal screening results for their latest child were excluded from the study, and it can take up to 5 weeks to process NBS results from all disorders of the entire program. If the newborn deceased in this period after birth, parents were not invited to participate. Parents' addresses were obtained via the National Institute for Public Health and the Environment (RIVM) after approval of working party Management Information System (MIS) of the RIVM. Parents were able to send back a printed questionnaire or to fill in the questionnaire online by following a link or scanning a QR-code. The survey was available in Dutch and accompanied by a cover letter from the RIVM with information about the study and privacy regulations. Filling out the questionnaire was voluntary and participation after receiving the invitation implied consent. All data was analyzed anonymously. Due to privacy reasons, no reminders were allowed to be sent. The study was approved by the Medical Ethics Committee of the Erasmus Medical Center, Rotterdam, the Netherlands (MEC-2017-1146).

### Questionnaire Design and Measures

A questionnaire about A-T was specifically developed for this study by a multidisciplinary group of experts on A-T, NBS, medical ethics, and survey studies. The questionnaire was based on the literature and questionnaires previously used for investigating parents' perspectives on NBS e.g., for Pompe disease (16). The questionnaire focused on the dilemma of early diagnosis of A-T and consisted of open questions with additionally multiple choices, scales, and yes/no answers. Since the disorder A-T is rare and parents are not acquainted with the symptoms and course of the disorder, the questionnaire started with a background information section on A-T (Supplementary Section A). The questionnaire consisted of four sections (Supplementary Sections B–E): (1) scenarios about early/late diagnosis of A-T, (2) statements about advantages and disadvantages of early diagnosis A-T, (3) final questions with decisive arguments, and (4) sociodemographic questions. A small test phase was conducted to check for concept and wording of questions. The questionnaire has 23 questions in total and took ~20 min to complete.

The scenarios included two cases of children with A-T: one with a late diagnosis of A-T at the age of 4 years and one with an early diagnosis of A-T at birth as a result of NBS for SCID (Supplementary Section B). Parents were asked to list the advantages and disadvantages of both scenarios from their perspective in a free text response. The open questions were analyzed by dividing the answers into categories using a dichotomous variable scoring system. Answers could be assigned to multiple categories. Open questions were categorized independently by two different researchers to enhance the internal validity (MB and MH).

The scenarios were followed by eleven statements about advantages of early detection and nine statements about disadvantages of early detection of A-T (Supplementary Section C). Parents could indicate their degree of support on a five-point Likert scale (1 = totally disagree to 5 = totally agree). Two statements were added to the questionnaire that were also included in the study of Schoenaker et al. (17) that aimed to investigate the perspective of A-T families on early detection of A-T. This way, a comparison could be made to the perspective of parents of A-T patients. Parents were additionally asked to indicate their degree of support on a five point scale (1 = totally disagree to 5 = totally agree) about the current follow-up policy after an abnormal NBS result for SCID. The statements included “In the case of an abnormal SCID screening result, diagnostics for A-T should be applied immediately” and “In the case of an abnormal SCID screening result that turns out not be SCID after follow-up diagnostics, diagnostics for A-T should not be applied. Additional diagnostics for A-T should only be used if symptoms of A-T begin to occur.”

The final questions included two hypothetical questions (Supplementary Section D). The first question “If a test would be available to screen all newborns for A-T, would you personally participate in this screening?” had a five point scale answer (1 = yes, 2 = probably yes, 3 = don't know, 4 = probably no, 5 = no). Parents were asked to choose their decisive arguments from multiple answers. The decisive argument to use or not use a hypothetical screening test for A-T was considered valid only if the respondent had a matching yes/probably yes or no/probably no answer. If the respondent noted more than one decisive argument, the answer was coded as “other.” The second question “Do you think A-T should be included in the NBS program?” could be answered on a three point scale (1 = yes, 2 = don't know, 3 = no).

The questionnaire ended with a sociodemographic section that included questions about gender, age, ethnicity, and educational level. Respondents were asked to indicate the highest level of education they had completed. Education level was grouped into three categories: low, middle and high (see Table 1). Ethnicity was coded as “Dutch” or “Other” based on the country of birth and country of birth of mother and father. Due to underrepresentation of the non-Dutch group, no distinction was made between Western and non-Western background. Furthermore, parents were asked to fill in the number of children they have/had, including their age, NBS results and health status. Civil registry status “single” and NBS parameters “not participated” and “abnormal screening results” were strongly underreported in the study population, therefore the relationship between variables and attitude toward early detection A-T was not analyzed.

**Table 1:** Sociodemographics of the respondents.

**Variables**	**Research population *N* = 659**	**Reference group** **Dutch population *N* (x1000)**	***p*-value**
Age in years (SD)		Dutch parents^a^	
Mean age of mothers in research/reference population	34.7 (4.81)	34.2	0.341
Mean age of fathers in research/reference population	32.1 (4.22)	31.3	<0.001
Gender, *n* (%)		Dutch population age 20–50 years^b^	<0.001
Male	86 (13.1)	3 304 (50.3)	
Female	571 (86.9)	3 266 (49.7)	
Missing	2		
Ethnicity, *n* (%)		Dutch population age 20–50 years^c^	<0.001
Dutch	569 (86.9)	4 675 (70.6)	
Other	86 (13.1)	1 932 (29.4)	
Missing	4		
Civil registry, *n* (%)		Dutch parents^d^	<0.001
Single	19 (2.9)	572 (21.6)	
Living together/married	637 (97.1)	2 024 (78.4)	
Missing	3		
Highest education level, n(%)^e^		Dutch population age 25–45 years^e^	<0.001
Low	24 (3.7)	585 (30.9)	
Middle	143 (21.8)	1643 (38.1)	
High	490 (74.6)	1908 (29.4)	
Missing	2		
Number of children, *n* (%)		Dutch parents^f^	0.0149
1	324 (49.5)	71.9 (44.2)	
2	219 (33.5)	62.5 (38.5)	
≥3	111 (17.0)	28.1 (17.3)	
Missing	5		

*Missing values were excluded from the percentages*.

a Reference population Dutch Parents (18). One sample t-test.

b Reference population Dutch population age 20–50 years (19). χ^2^ test.

c Reference population Dutch population age 20–50 years (19). χ^2^ test.

d Reference population Dutch population households (20). χ^2^ test.

Low: primary education, lower vocational education, lower, and middle general secondary education.

Middle: middle vocational education, higher secondary education, and pre-university education.

High: higher vocational education and university.

Reference population Dutch population age 25–45 years (21). χ^2^ test.

f *Reference population Dutch parents (20). χ^2^ test for trend*.

### Statistical Analysis

Statistical analysis was carried with SPSS version 25.0 for Windows (SPSS, Inc., Chicago, IL, USA). Sociodemographic characteristics of participants were compared to the Dutch reference population reported by Statistics Netherlands with one sample-*t*-test for age, chi square test for trend for ordered categories and Pearson's chi square test for other characteristics. Descriptive statistics were used to describe characteristics of the respondents. Descriptive statistics were additionally used to determine frequencies of answers of participants categorized as dichotomous variables. Ordinal variables from scaled items are reported as means. Missing data in the study did not exceed 5% in any measure. For multivariate logistic regression analyses, items consisting of five-point scales were summarized to three-point scales: 1 = (totally) disagree, 2 = do not disagree/do not agree, and 3 = (totally) agree. Multivariate logistic regression analysis was performed to determine whether the variables, age, gender, ethnicity, educational level were associated with the “if a test was available to screen all newborns for A-T, I would participate” and “if a test was available, A-T should be added to newborn screening program.” Having one child, having a child with a (genetic) condition and having a family member with a hereditary disorder were included as variables as well. Standardized regression coefficients (β) are reported as an expression of the strength of the associated variables. Missing data were not analyzed in regression analyses. *P*-values <0.05 were considered statistically significant.

## Results

### Response and Demographics

A total of 664 of 4,000 parents sent back the questionnaire leading to a response rate of 16.6%. The majority of parents responded by sending the printed questionnaire back (*n* = 550/82.8%) compared to 114 (17.2%) parents who filled in the questionnaire online. Questionnaires where at least the statements about disadvantages and advantages of early diagnose A-T were completed (Supplementary Section C), were considered eligible for analysis. Based on this criterion, five questionnaires were excluded from the study resulting in the analysis of 659 questionnaires.

The respondents' characteristics are given in Table 1. The mean age of respondents was 32.4 years (range 20–47 years). Women were overrepresented in the respondent group (86.9%). Compared to the reference population, the respondents were more highly educated and more likely to have a Dutch ethnic background (19, 21). The average number of children was 1.73 (range 1–11 children) compared to 1.61 in the reference population of Dutch parents (20) (Table 1). The vast majority of parents (99.5%) indicated that all their children had participated in the Dutch NBS program. Of the five parents that indicated that one of their children had not participated, all stated that newborn screening was performed abroad. As expected, parents reported that most NBS results were normal. Abnormal results included congenital hypothyroidism (*n* = 1) and carrier status of sickle cell anemia (*n* = 1). Twenty-four parents with a child with a (genetic) condition mentioned a range of hereditary disorders whereas participations who indicated the presence of a family member with a hereditary disorder (17.2%) mentioned a broad spectrum of as well disorders (Table 2).

**Table 2:** Participation NBS, health status of the children and familial hereditary disorders.

	**Research population**	
	***N* = 659**	**%**
**Did all your children participate in the dutch NBS program?**		
Yes	644	99.5
No	5	0.5
Missing	10	
**What was the NBS result for your child (-ren)?**		
Normal	643	99.5
Abnormal	2	0.3
I'd rather not say	1	0.2
Missing	13	
**Are your children healthy?**^**a**^		
Yes	628	96.0
No	24	3.7
I'd rather not say	2	0.3
Missing	5	
**Do you have a family member with a hereditary disorder?**^**b**^		
Yes	112	17.2
No	501	76.8
I don't know	35	5.4
I'd rather not say	4	0.6
Missing	7	

*Missing values were excluded from the percentages*.

a Answers included a wide variety of hereditary disorders including Down Syndrome, Fragile X-syndrome, metabolic diseases, and diabetes mellitus type 1.

b *Answers included a broad spectrum of disorders such as malignancies, diabetes mellitus, cardiovascular diseases, and autoimmune diseases*.

### Attitude Toward Late and Early Detection of A-T

In total, 652 out of 659 parents listed advantages and disadvantages to the scenarios about late and early detection of A-T (Table 3). The majority of parents (57.1%) indicated the “golden/happy” years, the asymptomatic years without worries or anxiety, as the main advantage of late diagnosis of A-T. In addition, parents mentioned that it would be an advantage for the child to not receive medical labeling from birth, allowing them to develop at their own pace. Other advantages mentioned were: the opportunity to fully enjoy the maternity period (10.3%) and the ability to have another child without any worries about the disease (8.5%). Even though parents were asked to indicate the advantages of late detection, more than a quarter of parents stated that they did not see any advantages of late detection of A-T (26.1%). The main disadvantage of late detection of A-T in the perspective of parents was linked to the hereditary character of the disorder (46.2%). The case described the situation in which the couple already had a second child when their first child was diagnosed with A-T. Not being able to make a well-informed decision about family planning or prenatal diagnostics was an important negative aspect for parents. Parents also associated late diagnosis of A-T with a delayed start of medical access (guidance and surveillance of the patient and family) (42.6%) a long period of uncertainty and worries (30.8 and 21.5%) and delayed breast cancer screening for the mother of the A-T patient (18.4%). One eighth of the parents (12.8%) additionally mentioned not being able to mentally or financially prepare for the diagnosis as a disadvantage.

**Table 3:** Advantages and disadvantages of late and early detection of A-T according to parents (*n* = 652 respondents).

**Late detection A-T**		**Early detection A-T**	
**Advantages**	**Disadvantages**	**Advantages**	**Disadvantages**
Carefree period (57.1%)	Heredity (chance of another child with A-T) (46.2%)	Start with supportive treatment (49.2%)	No worry-free period (48.9%)
Parents who stated they saw no advantages in late detection of A-T (26.1%)	Delayed start of treatment/surveillance (42.6%)	Clarity, knowing what to expect (35.6%)	Unable to enjoy the maternity period (47%)
No medical labeling of child (11.2%)	Long period of uncertainty (30.8%)	Surveillance by specialists (37.7%)	The baby has no symptoms yet (23.2%)
Being able to fully enjoy the maternity period (10.3%)	Long period of worries (21.5%)	Early breast cancer screening mother (27.2%)	Devastating news in a mentally emotional period (15.1%)
Being able to make a carefree choice to have another child (8.5%)	Delayed breast cancer screening mother (18.4%)	Being able to prepare (mentally/practically) for a sick child (26.3%)	Insecurity about the future (14.3%)
	No time to prepare (mentally/practically)/ make adjustments in your life (12.8%)	Being able to make informed reproductive choices (13.1%)	Difficulty to process information directly after birth (8.7%)

The main advantage of early detection of A-T from a parents' perspective was the ability to start with supportive treatment (e.g., physiotherapy) and receiving the most optimal clinical guidance right from the start (49.2%). Surveillance by a multidisciplinary team of specialists was mentioned by 37.7% of the parents as well. Parents highly valued clarity and knowing what to expect in contrast to the uncertainty and insecurity that are accompanied by a late diagnosis of A-T. Other advantages mentioned were: early breast cancer screening for the mother of the A-T patient (27.2%), the ability to (mentally and practically) prepare for a life with a child with a serious condition (26.3%) and the opportunity to make an informed reproductive choice (13.1%). The exclusion of a worry- or care-free period (48.9%) next to the inability to enjoy the maternity period (47%) were listed by parents as the main disadvantages of early detection of A-T. These disadvantages were directly linked to the difficulty to process such devastating news in an emotional and hormonal period after birth (15.1 and 8.7%). Other disadvantages of early detection of A-T mentioned were: the asymptomatic newborn (“the baby has no symptoms yet”) (23.2%) and the insecurity with regard to the future (14.3%). In general, parents were able to indicate more advantages for early detection than for late detection of A-T. Several parents mentioned the difficulty of the dilemma and the ability to argue for both sides.

### Level of Agreement With Regard to Advantages and Disadvantages Early Diagnosis A-T

Parents were asked to indicate their level of agreement of support for eleven statements about advantages of early detection and nine statements about disadvantages of early detection of A-T. The statement with the highest level of support indicated that parents value the fact that an early diagnosis of A-T will ensure that a child with A-T will immediately receive optimal guidance when the first symptoms occur (rating mean 4.5) (Table 4). Additionally, most parents agreed that early diagnosis of A-T would prevent a long period between the first symptoms and eventual diagnosis (rating mean 4.2) and with that, a long time of uncertainty for parents (rating mean 4.2). Family planning, early breast cancer screening for mothers and the opportunity to make adjustments into your lives were all advantages of early diagnosis A-T parents agreed with. In contrast, saving extra health associated costs and the idea that parents will be able to take better care of their child if diagnosed early, do not show the same levels of support (both rating mean 3.3). Parents perceive the most important disadvantages of early detection of A-T as “early detection of A-T overburdens parents with information about an untreatable disease during the maternity period” and “early detection of A-T deprives parents of the opportunity to enjoy a seemingly healthy baby in the first months/years of life (both rating mean 3.4) (Table 5). Other disadvantages were met with neutrality or disagreement. For most parents, the fact that A-T cannot be cured or treated is not perceived as a disadvantage of early detection (rating mean 2.5). Arguments as “taking life as it comes” (rating mean 2.5) or “early detection will reduce the bond between parents and child (rating mean 2.5) were not agreed with. Several parents mentioned that they agreed with the statements about late detection of A-T, but that they see more benefits in early detection of A-T.

**Table 4:** Level of agreement with regard to advantages of early detection of A-T.

**Survey question:**	**Level of agreement**^**a**^ **(%)**					**Rating mean (SD)**
	**Fully** **disagree**				**Fully** **agree**	
Early detection of A-T ensures that a child with A-T can immediately receive optimal guidance when the first symptoms occur	1.7	1.6	0.8	34.3	61.6	4.5 (0.75)
Early detection of A-T prevents a long period between the first symptoms and the eventual diagnosis	1.9	3.7	7.9	47.0	39.4	4.2 (0.87)
Early detection of A-T provides parents with the opportunity to make informed choices about family planning	2.8	3.3	5.8	43.1	45.0	4.2 (0.91)
Early detection of A-T prevents a long period of uncertainty for parents	3.1	5.3	6.9	40.1	44.2	4.2 (0.99)
Early detection enables parents to make early adjustments into their lives (for example wheelchair accessible house)	2.0	6.2	13.1	49.9	28.1	4.0 (0.92)
It is an advantage that parents are informed about the slightly increased risk of developing breast cancer for the mother	2.5	5.0	12.3	45.2	34.2	4.0 (0.95)
Early detection of A-T ensures that parents can adjust their expectations about the condition of their child	2.3	7.3	10.0	50.9	29.0	4.0 (0.95)
Early detection of A-T prevents unnecessary additional tests	1.9	7.8	14.2	51.5	24.3	3.9 (0.93)
Early detection of A-T prevents multiple visits to the hospital	2.8	15.6	20.3	42.5	20.3	3.6 (1.05)
Early detection of A-T saves extra health costs	6.1	17.9	26.4	36.3	12.6	3.3 (1.10)
Early detection of A-T ensures that parent can take better care of their child	10.0	17.2	25.6	27.6	19.0	3.3 (1.24)

*SD, Standard deviation. ^a^Five-point rating scale: 1, fully disagree; 5, fully agree; n = 659 respondents. Missing values are excluded from the percentages*.

**Table 5:** Level of agreement with regard to disadvantages of early detection of A-T.

**Survey question:**	**Level of agreement**^**a**^ **(%)**					**Ratings mean (SD)**
	**Fully** **disagree**				**Fully** **agree**	
Early detection of A-T overburdens parents with information about an untreatable disease during the maternity period	7.3	19.7	13.4	42.4	16.4	3.4 (1.19)
Early detection of A-T deprives parents of the opportunity to enjoy a seemingly healthy baby in the first months/years of life	5.5	20.7	18.3	38.4	16.5	3.4 (1.15)
Early detection of AT makes parents worry about the disease before the symptoms even occur	9.0	27.9	15.8	38.4	8.1	3.0 (1.16)
Every child has the right to an open future	11.1	24.3	31.2	21.5	10.6	3.0 (1.16)
Early detection of A-T overburdens parents with information about the increased risk of breast cancer for the mother during the maternity period	10.6	33.5	15.6	30.0	9.4	2.9 (1.20)
Early detection of A-T adds little to the quality of life of a child with A-T	12.6	44.9	20.0	17.2	4.7	2.6 (1.06)
The disease A-T cannot be prevented or treated anyway	19.8	37.8	18.1	18.4	4.7	2.5 (1.14)
You have to take life as it comes	19.8	31.5	28.2	14.5	5.0	2.5 (1.06)
Early detection of A-T can lead to a reduced bond between parents and child	38.7	29.5	15.4	11.5	4.5	2.1 (1.18)

*SD, Standard deviation. ^a^Five-point rating scale: 1, fully disagree; 5, fully agree; n = 659 respondents. Missing values are excluded from the percentages*.

### Intention to Participate in A-T Screening and Opinion on Current Policy for NBS for SCID

In total, 288 of the parents (44%) would participate in A-T screening if a test would be available (as they indicated “yes” to this hypothetical question). In addition, 234 of the parents (37.1%) intended to participate in screening for A-T if a test would be available (indicated by “probably yes”). The two main decisive arguments to participate were: “early detection of A-T prevents a long period between the first symptoms and the diagnosis” and “early detection of A-T ensures that a child with A-T can immediately receive optimal guidance when the first symptoms occur.” In total, 16 parents (2.4%) did not intend to participate in screening for A-T. Moreover, 47 parents (7.2%) would probably not participate in screening for A-T. The main decisive argument to decline screening for A-T was: “early detection of A-T deprives parents of the opportunity to enjoy a seemingly healthy baby in the first months/years of life.” In the case of an abnormal screening result for SCID, 81.9% (*n* = 538) of the parents think that diagnostics for A-T should be applied. In addition, the majority of parents (72.9%/*n* = 478) disagrees with the current NBS for SCID protocol in which A-T diagnostics are not applied after abnormal SCID screening results, but only if symptoms of A-T start to occur. The opinion of parents of A-T patients, as described recently by Schoenaker et al. (17) did not differ from our research population with regard to this policy (*p* = 0.403). Parents of A-T patients were less convinced that A-T should be added to the NBS program if a test was available in comparison to parents of healthy newborns (76 vs. 91.4%, respectively) (Table 6).

**Table 6:** Comparison to the perspective of parents of A-T patients: opinions on current policy and NBS for A-T.

**Survey question:**	**Parents of A-T patients** **Degree of support**^**a**^ ***n*** **(%)** **Total** ***n*** **=** **35**^**b**^					**Parents of healthy newborns** **Degree of support**^**a**^ ***n*** **(%)** **Total** ***n*** **=** **659**					***p*-value^**c**^**
In the case of an abnormal SCID screening result that turns out not be SCID after follow-up diagnostics, diagnostics for A-T should not be applied. Additional diagnostics for A-T should only be used if symptoms of A-T begin to occur.	Fully disagree			Fully agree		Fully disagree			Fully agree		0.403
	12 (37.5)	12 (37.5)	3 (8.8)	6 (17.4)	1 (2.9)	150 (22.9)	328 (50.0)	61 (9.3)	90 (13.7)	27 (4.1)	
	Missing 1					Missing 3					
If a technique was available that would be able to detect all children with A-T with NBS, A-T should be included in the NBS program.	No			Yes		No			Yes		0.003
	8 (24%)			25 (76%)		49 (8.6%)			523 (91.4%)		
	Missing 2					Missing 10^d^					

*SD, Standard deviation*.

a Five-point rating scale: 1, fully disagree; 5, fully agree; Missing values are excluded from the percentages.

b Data collected via the questionnaire sent to parents of A-T patients (17).

c χ^2^ test.

d *n = 77 answered “don't know” and were excluded from analysis*.

### Multivariate Logistic Regression Regarding Newborn Screening for A-T

The only variable with a significant association to the outcome variables was “the number of children” (Table 7). Respondents who had their first child (number of children 1) were more likely to participate in NBS for A-T than respondents with more children (number of children >1). Parents with one child were also more likely to believe that A-T should be added to the NBS program. Other variables [age, gender, ethnicity, level of education, having a child with a (genetic) condition and having a family member with a hereditary disorder] were not significantly associated with any of the outcome variables (Table 7).

**Table 7:** Multivariate logistic correlation.

**Predictor variable**	**A-T should be added to NBS program**			**Intended participation NBS A-T**		
	**B**	**SE**	***p*-value**	**B**	**SE**	***p*-value**
Age (20–30 years)	1.001	0.617	0.105	−0.205	0.608	0.736
Gender (female)	0.409	0.488	0.402	−0.137	0.409	0.738
Ethnicity (Dutch)	−1.327	0.743	0.74	1.090	0.612	0.075
Educational level (high)	−0.11	0.382	0.977	−15.927	1929.242	0.736
Number of children (first child)	17.173	0.623	0.0001	16.097	0.653	0.0001
Having a sick child (yes)	0.480	0.786	0.374	0.138	0.781	0.860
Having a family member with a hereditary disease (yes)	−15.998	5102,717	0.997	16.322	5405.408	0.998

*Multivariate logistic regression analyses (n = 581 valid cases) with standardized regression coefficients β and standard error (SE). Missing values were excluded from the multivariate regression analysis*.

## Discussion

The aim of this study was to provide insight into parents' perspectives about the early detection of A-T and with that to collect empirical data on public acceptance of untreatable findings to NBS. The vast majority of parents in our study population believed that advantages of early detection of A-T outweighed the disadvantages (81.9%). The prevention of a long period between first symptoms and diagnosis and the fact that early detection will ensure that a child with A-T can immediately receive optimal guidance when the first symptoms occur were the most important arguments from their perspective. Parents who see more disadvantages than advantage in early detection of A-T (9.6%) believe that early detection of A-T deprives parents of the opportunity to enjoy an apparent healthy baby in the first months/years of life. The public attitude toward reporting A-T as an untreatable incidental finding of NBS for SCID thus appeared to be positive. In the case of an abnormal screening result for SCID, 81.9% of the parents think that diagnostics for A-T should be applied. In addition, the majority of parents (72.9%) disagree with the current NBS for SCID protocol in which A-T diagnostics are not applied after abnormal SCID screening results, but only if symptoms of A-T start to occur.

The perspective of parents of healthy newborns is a reflection of the public, but the opinions of parents of patients are of great importance as well. Both parents of healthy newborns and parents of A-T patients favored the advantages of early detection of A-T in the asymptomatic phase over the disadvantages (17). Decisive arguments differed amongst groups; whereas parents of healthy newborns valued the optimal clinical guidance from the start, parents of a child with A-T mentioned the uncertainty toward the diagnosis and the impact on their lives. This last argument would be difficult to envision for parents of healthy newborns, as they have not experienced it first-hand. Parents of A-T patients additionally mentioned the importance of knowledge about the inheritance and recurrent risk of A-T when making reproductive choices (17). Both parents of healthy newborns and parents of A-T patients who were opposed to early detection of A-T valued the “happy/golden years.” These findings suggest that first-hand experience with the untreatable disorder is an independent factor in the final opinion of parents on early detection of this disorder, although the arguments used are colored by these experiences.

The discussion about reporting untreatable incidental findings goes hand in hand with the discussion about NBS for untreatable disorders. There is a difference in actively screening for untreatable disorders and reporting them as incidental findings to NBS for treatable disorders. In this study, both aspects were studied amongst parents: the situation of A-T as untreatable disorder to NBS for SCID discussed previously and the hypothetical situation of NBS for A-T. The result showed high support for neonatal screening for A-T in the general public. The support was consistent for both the public health perspective (should A-T be added to the neonatal screening program?) and the personal perspective (would you use the screening?). The great majority of parents would (probably) participate in NBS for A-T if a test would be available. Moreover, most parents were convinced that A-T should be added to the NBS program if a test was available. These findings are in direct contradiction to the Wilson and Jungner criteria (1968) which state the screened disorders should have an available treatment. Remarkably, results indicated that parents with (only) one child were more likely to participate in NBS for A-T than respondents having more children. This group was also more likely to believe that A-T should be added to the NBS program. These findings suggest that “new” parents have a higher support for NBS for A-T than parents with children who are somewhat older and are likely to be more experienced in parenting. A possible explanation could be that feelings of uncertainty that are accompanied with new parenthood, makes parents look for ways of health confirmation, such as participation in additional screening programs (22).

In addition to parents of healthy newborns, the majority of parents of A-T patients were in favor of adding A-T to the NBS program as well. This implies a high level of support for NBS for A-T, not only among those who have personal experience of the disease but also among the general public. In the past, patient organizations have promoted the expansion of NBS for particular conditions, while evidence-based reviews by professional experts have been more hesitant (23). Our findings are similar to studies about NBS for other (previously considered as) untreatable disorders. The study of Weinreich et al. (16) compared the perspective of a consumer panel with (parents of) patients with Pompe disease. In total, 87% of the consumer panel and 88% of the Pompe group supported the introduction of NBS for Pompe (16). The study of Wood et al. (24) showed high support amongst parents of children with Duchenne and Becker Muscular Dystrophy and Spinal Muscular Atrophy (SMA) for NBS for these conditions. Of their survey cohort, 95.9% of believed that NBS should be implemented, even in the absence of therapeutic consequences (24). These findings can also be extrapolated to the opinion of the general public. In the United Kingdom, a survey study revealed that 84% of participants from the general public were in favor of NBS for SMA, compared to 70% support among SMA families (25). In the meantime, treatment for SMA became available and in July 2019, the Health Council of the Netherlands deemed SMA to be a suitable candidate to be included in the Dutch NBS program (26). Focus groups amongst a diversity of mothers with young children showed great support for NBS for untreatable conditions presenting in infancy. Similar arguments to our study population were mentioned such as the importance of emotional preparation and the avoidance of the “diagnostic Odyssey” (27). Furthermore, in the study of Hayeems et al. (28) the majority of participations in focus groups supported NBS for serious disorders for which treatment is not available (95–98, 82%).Anticipated benefits of expanded infant screening were prioritized over harms (28). However, the authors urged caution around the potential for public enthusiasm to foster unlimited uptake of infant screening technologies.

The perspective of parents as key stakeholders in NBS is of great value for policymaking. While some countries embrace all incidental findings, the current policy in the Netherlands on reporting untreatable incidental findings is more conservative. Cultural and moral believes seem to be of influence in the decision making process around screening and reporting of untreatable (incidental) findings. Expanding our study to other countries who have implemented NBS for SCID would create an interesting opportunity to study the influence of these believes on parents' perspective on screening for untreatable disorders. Policy makers need to balance different perspectives and needs in discussion about NBS for untreatable disorders, such as high quality evidence, benefits or harms for the routine screening program, costs, values of the population as well as contextual considerations. The Health Council of the Netherlands stated in 2015 that some benefits of screening/reporting for untreatable (incidental) disorders such as shortening the diagnostic process and the ability to adapt/prepare to a life with a condition might be in the interest of the child. In addition, a long-term diagnostic process can have negative effects on the psychological well-being of a child and his or her family (15). However, as it is not self-evident that screening for untreatable disorders is in the best interest of the child and as empirical data on the advantages and disadvantages of early knowledge of untreatable disorders are limited, the discussion in the Netherlands is ongoing. Without scientific evidence that neonatal screening can prevent significant health damage, the Council states that extending the NBS program with untreatable diseases would be undesirable (15). Our results as well as other studies that showed support for the screening of untreatable disorders will serve as valuable tools and scientific evidence in advising policymakers in their considerations about NBS for non-treatable disorders.

This study encountered several strengths and limitations. The questionnaire was sent to a large number of parents thereby increasing the external validity of the study. Moreover, the use of a sequential mixed methods approach and open coding by two different researchers (MB and MH) increased the internal validity and enhanced a deeper understanding of the subject. The ability to compare our study data of parents of healthy newborns with the data of parents of A-T patients (17) provides a complete overview of the perspective of different groups of parents on the early detection of A-T in a pre-symptomatic phase. In additions to these strengths, the study has some limitation. The research population is significantly different from the Dutch reference population and may therefore not completely reflect the attitude of the general Dutch population. Some parents indicated that the questions could be experienced as too difficult which could result in bias toward higher educated respondents. In addition, the participants in the study were chosen among those who voluntary participated in the SONNET study. This could potentially create a study population biased toward favoring NBS for any disorder. As the objection rate in the SONNET-study was only 0.6% (data not published), bias is limited and the results of this questionnaire study would still reflect the perspective of the majority of parents. Finally, the study has a relatively low response rate. Previous studies indicate that a low response rate does not automatically mean the study results have low validity (29), they simply indicate a potentially greater risk of this. This study reports methods of recruitment and provides detailed information about the respondents increasing the validity and utility of the study results. The response rate could be improved if a reminder was allowed to be sent (30).

## Conclusion

Reporting untreatable incidental findings remains a disputed topic worldwide. The current policy in the Netherlands is to not report these incidental findings, unless early detection prevents significant health damage to the child. The majority of parents of healthy newborns are in favor of an early A-T diagnosis in the pre-symptomatic phase of the disease. Moreover, the majority of parents would use a screening test for A-T, if such a test were available. Decisive arguments to participate were the fact that early detection of A-T prevents a long period between the first symptoms and the diagnosis and that early detection of A-T ensures immediate optimal guidance for a child when the first symptoms occur. With the ongoing discussion in the Netherlands on reporting untreatable incidental findings and NBS for untreatable diseases, parent's perspective could be used as a valuable tool for policy makers who aim to balance advantages and disadvantages of early detection of rare hereditary disorders.

## Data Availability Statement

The datasets generated for this study are available on request to the corresponding author.

## Ethics Statement

This study involving human participants was reviewed and approved by the Medical Ethics Committee of the Erasmus Medical Center, Rotterdam, the Netherlands (MEC-2017-1146). Written informed consent for participation was not required for this study in accordance with the national legislation and the institutional requirements.

## Author Contributions

MBl, MS, and MBu designed the study. MBl, MV, CW, MW, and LH developed the questionnaire. MBl and MH collected and analyzed the data. MBl, MH, and MBu wrote the paper. All authors edited the paper and approved the final version.

### Conflict of Interest

The authors declare that the research was conducted in the absence of any commercial or financial relationships that could be construed as a potential conflict of interest.
