# Supplementary material for: Dilemma of Reporting Incidental Findings in Newborn Screening Programs for SCID: Parents’ Perspective on Ataxia Telangiectasia
Source: Front Immunol. 2019 Nov 6;10:2438. doi: 10.3389/fimmu.2019.02438 (PMC6851017; doi:10.3389/fimmu.2019.02438)
Supplement: Supplementary file 1 [file Data_Sheet_1.PDF]

Questionnaire
Early diagnosis of Ataxia Telangiectasia

This questionnaire consists of five different sections:

- Section A. Background information about Ataxia Telangiectasia (A-T)
- Section B. Scenarios of two A-T patients
- Section C. Statements about early detection of A-T
- Section D. Final questions
- Section E. Demographics

A. Background information

Ataxia Telangiectasia (A-T) is a rare, serious disease. This disease causes serious, progressive neurological symptoms, such as problems with balance and coordination. Patients with A-T have more frequent infections and an increased risk of developing cancer, in particular of the blood and lymph nodes (leukemia or lymphomas). Children with A-T have a shorter life expectancy. In addition, mothers of A-T patients have a slightly increased risk of developing breast cancer. A-T is a hereditary disease. This means that other children within the same family could also have A-T.

There is no cure for A-T and it is not possible to delay the disease-onset or progression. The treatment is aimed at treating the various symptoms of the disease. For example, children with A-T often receive physiotherapy and come to the hospital for a check-up with the pediatrician once a year. As A-T is a rare disease and not all characterizing symptoms might be present in each case, it sometimes takes doctors quite some time to think about the diagnosis A-T.

VL2

SONNET

B. Scenarios and questions

If a newborn has an abnormal newborn screening result for SCID, the newborn will be referred for additional confirmatory diagnostics in the (academic) hospital. An abnormal screening result means that the child might have SCID. The child could also have another disease with a immune disorder such as A-T. Children with A-T are asymptomatic at birth. In this section, two stories of children with A-T are described, followed by questions.

- Early diagnosis of A-T or early detection of A-T means that the diagnosis A-T is directly made after birth. The child has no symptoms at that time.
- Late diagnosis of A-T or late detection of A-T means that the diagnosis A-T is made later in life when the child has already developed symptoms.

Scenario 1. Max and a late diagnosis of A-T

Max is three days old when newborn screening is performed. The newborn screening result for SCID is abnormal. A pediatrician in the hospital performs additional medical examinations. Max does not have SCID. No additional diagnostics is done for Ataxia Telangiectasia and Max is allowed to go home. When Max is 12 months old, he has difficulty with crawling. The youth healthcare doctor thinks that Max is just developing a little bit slower compared to his age group. When Max is 20 months old, he starts walking, but falls over a lot. He also often has colds. Max's mother is worried. The GP thinks that Max's walking will improve in the future. When Max continues to fall over and his walking does not improve, Max's parents are referred to the hospital. Max is 4 years old by that time and now has a little brother of 6 months old. The pediatrician performs additional diagnostics and diagnoses Max with Ataxia Telangiectasia (A-T). Max's parents are told that A-T cannot be cured. Max can end up in a wheelchair and there is a chance that he will develop cancer at a young age. His life expectancy is shorter compared to other childern. Max's mother is referred for early breast cancer screening. Additional diagnostics are being done to test if Max's little brother also has A-T.

1. What do you believe to be the advantages of late detection of A-T in Max's case?

2. What do you believe to be the disadvantages of late detection of A-T in Max's case?

Scenario 2. Lotte and an early diagnosis of A-T

Lotte is three days old when newborn screening is performed. The newborn screening result for SCID is abnormal. A pediatrician in the hospital performs additional medical examinations. Lotte does not have SCID. Additional diagnostics are done for Ataxia Telangiectasia. After three weeks, Lotte's parents receive the results of the additional tests: Lotte has Ataxia Telangiectasia. Lotte is a seemingly healthy baby girl without symptoms at the time of diagnosis. A pediatrician explains to Lotte's parents that A-T cannot be cured. Lotte can end up in a wheelchair and has a shorter life expectancy compared to other children. Lotte's mother finds it difficult to process all this information. She is still recovering from child-birth. Since Lotte currently has no symptoms, physical therapy will not yet be started. However, from now on Lotte will be closely monitored by an experienced team of medical specialists. This team of specialist will immediately check-up on Lotte when the first symptoms of A-T occur. Lotte's mother is referred for early breast cancer screening.

3. What do you believe to be the advantages of early detection of A-T in Lotte's case?

4. What do you believe to be the disadvantages of early detection of A-T in Lotte's case?

C. Statements about early detection of A-T

- Early diagnosis of A-T or early detection of A-T means that the diagnosis A-T is directly made after birth. The child has no symptoms at that time.
- Late diagnosis of A-T or late detection of A-T means that the diagnosis A-T is made later in life when the child has already developed symptoms.

CI. A number of advantages of early detection of A-T are stated below. Please indicate which box reflects your opinion best.

A-T should be detected early, because:

	Fully disagree	Disagree	Neither agree, nor disagree	Agree	Fully agree
Early detection of A-T prevents a long period between the first symptoms and the eventual diagnosis	☐	☐	☐	☐	☐
Early detection of A-T prevents multiple visits to the hospital	☐	☐	☐	☐	☐
Early detection of A-T saves extra health costs	☐	☐	☐	☐	☐
Early detection of A-T prevents a long period of uncertainty for parents	☐	☐	☐	☐	☐
Early detection of A-T prevents unnecessary additional tests	☐	☐	☐	☐	☐
Early detection of A-T provides parents with the opportunity to make informed choices about family planning	☐	☐	☐	☐	☐
Early detection of A-T ensures that a child with A-T can immediately receive optimal guidance when the first symptoms occur	☐	☐	☐	☐	☐
It is an advantage to be informed about the slightly increased risk of developing breast cancer for mother	☐	☐	☐	☐	☐
Early detection of A-T ensures that parents can adjust their expectations about the condition of their child	☐	☐	☐	☐	☐
Early detection enables parents to make early adjustments into their lives (for example wheelchair accessible house)	☐	☐	☐	☐	☐
Early detection of A-T ensures that parent can take better care of their child	☐	☐	☐	☐	☐

2. A number of disadvantages of early detection of A-T are stated below. Please indicate which box reflects your opinion best.

A-T <u>should not</u> be detected early, because:

	Fully disagree	Disagree	Neither agree, nor disagree	Agree	Fully agree
Early detection of A-T adds little to the quality of life of a child with A-T	☐	☐	☐	☐	☐
Early detection of A-T overburdens parents with information about an untreatable disease during the maternity period	☐	☐	☐	☐	☐
Early detection of A-T deprives parents of the opportunity to enjoy a seemingly healthy baby in the first months/years of life	☐	☐	☐	☐	☐
You have to take life as it comes	☐	☐	☐	☐	☐
Early detection of A-T overburdens parents with information about the increased risk of breast cancer for mother during the maternity period	☐	☐	☐	☐	☐
The disease A-T cannot be prevented or treated anyway	☐	☐	☐	☐	☐
Early detection of AT makes parents worry about the disease before the symptoms even occurred	☐	☐	☐	☐	☐
Early detection of A-T can lead to a reduced bond between parents and child	☐	☐	☐	☐	☐
Every child has the right to an open future	☐	☐	☐	☐	☐

4. Two statements are presented below. Please indicate which box reflects your opinion best.

Statement 1. In the case of an abnormal SCID screening result, diagnostics for A-T should be applied immediately

Fully disagree	Disagree	Neither agree, nor disagree	Agree	Fully agree
☐	☐	☐	☐	☐

Statement 2. In the case of an abnormal SCID screening result that turns out not be SCID after follow-up diagnostics, diagnostics for A-T should not be applied. Additional diagnostics for A-T should only be used if symptoms of A-T begin to occur

Fully disagree	Disagree	Neither agree, nor disagree	Agree	Fully agree
☐	☐	☐	☐	☐

D. Final questions

1. It is not yet possible to detect all patients with A-T with newborn screening. However, if a test would be available to screen all newborns for A-T, would you personally participate in this screening?

No	Probably not	Don't know	Probably yes	Yes
☐ Continue to question 2	☐ Continue to question 2	☐	☐ Continue to question 3	☐ Continue to question 3

If you have answered 'don't know', you can skip questions 2 and 3 and continue to question 4.

2. What would be the **decisive** argument for you to **not participate** in newborn screening for A-T?

- ☐ Early detection of A-T adds little to the quality of life of a child with A-T
- ☐ Early detection of A-T overburdens parents with information about an untreatable disease during the maternity period
- ☐ Early detection of A-T deprives parents of the opportunity to enjoy a seemingly healthy baby in the first months/years of life
- ☐ You have to take life as it comes
- ☐ Early detection of A-T overburdens parents with information about the increased risk of breast cancer for the mother during the maternity period
- ☐ The disease A-T cannot be prevented or treated anyway
- ☐ Early detection of AT makes parents worry about the disease before the symptoms even occur
- ☐ Early detection of A-T can lead to a reduced bond between parents and child
- ☐ Every child has the right to an open future
- ☐ Other, please specify:

3. What would be the **decisive** argument for you to **participate** in newborn screening for A-T?

- ☐ Early detection of A-T prevents a long period between the first symptoms and the eventual diagnosis
- ☐ Early detection of A-T prevents multiple visits to the hospital
- ☐ Early detection of A-T saves extra health costs
- ☐ Early detection of A-T prevents a long period of uncertainty for parents
- ☐ Early detection of A-T prevents unnecessary additional tests
- ☐ Early detection of A-T provides parents with the opportunity to make informed choices about family planning
- ☐ Early detection of A-T ensures that a child with A-T can immediately receive optimal guidance when the first symptoms occur
- ☐ It is an advantage that parents are informed about the slightly increased risk of developing breast cancer for the mother
- ☐ Early detection of A-T ensures that parents can adjust their expectations about the condition of their child
- ☐ Early detection enables parents to make early adjustments into their lives (for example wheelchair accessible house)
- ☐ Early detection of A-T ensures that parent can take better care of their child
- ☐ Other, please specify:

4. If a technique was available that would be able to detect all children with A-T with newborn screening, do you think A-T should be included in the newborn screening program?

- ☐ Yes
- ☐ No
- ☐ Don't know

E. Demographics

1. I am…

☐ Male
☐ Female

2. What is your age?

3. What is your *highest* level of education?

☐ None, primary school
☐ LBO, MAVO
☐ VMBO
☐ MBO, HAVO, VWO
☐ HBO, University
☐ Other:

4. What is your marital status?

☐ Single
☐ Living together/married
☐ Other:

5. In which country were you born?

6. In which country was your father born?

7. In which country was your mother born?

8. How many children do you have?

9. Please answer for each child:

	How old is your child today?	Was newborn screening performed in the Netherlands?	What were the results of the newborn screening program? Options: good/not good/I'd rather not say
1st child			
2nd child			
3rd child			
4th child			
5th child			

10. Are you children healthy? If no, please specify why not.

- ☐ Yes
- ☐ No:
- ☐ I'd rather not say

11. Do you have a family member with a hereditary disorder? If yes, please specify the disorder in question.

- ☐ No
- ☐ Yes:
- ☐ I don't know
- ☐ I'd rather not say

12. If you have any additional comments about the questionnaire, please leave them below:

Thank you for your cooperation!

SONNET
